# Covert Anti-Compensatory Quick Eye Movements during Head Impulses

**DOI:** 10.1371/journal.pone.0093086

**Published:** 2014-04-14

**Authors:** Maria Heuberger, Murat Sağlam, Nicholas S. Todd, Klaus Jahn, Erich Schneider, Nadine Lehnen

**Affiliations:** 1 German Center for Vertigo and Balance Disorders, Munich University Hospital, Munich, Germany; 2 Department of Neurology, Munich University Hospital, Munich, Germany; 3 Institute for Clinical Neurosciences; Munich University Hospital, Munich, Germany; 4 Brandenburg Institute of Technology, Cottbus - Senftenberg, Germany; University of Iowa, United States of America

## Abstract

**Background:**

Catch-up saccades during passive head movements, which compensate for a deficient vestibulo-ocular reflex (VOR), are a well-known phenomenon. These quick eye movements are directed toward the target in the opposite direction of the head movement. Recently, quick eye movements in the direction of the head movement (covert anti-compensatory quick eye movements, CAQEM) were observed in older individuals. Here, we characterize these quick eye movements, their pathophysiology, and clinical relevance during head impulse testing (HIT).

**Methods:**

Video head impulse test data from 266 patients of a tertiary vertigo center were retrospectively analyzed. Forty-three of these patients had been diagnosed with vestibular migraine, and 35 with Menière’s disease.

**Results:**

CAQEM occurred in 38% of the patients. The mean CAQEM occurrence rate (per HIT trial) was 11±10% (mean±SD). Latency was 83±30 ms. CAQEM followed the saccade main sequence characteristics and were compensated by catch-up saccades in the opposite direction. Compensatory saccades did not lead to more false pathological clinical head impulse test assessments (specificity with CAQEM: 87%, and without: 85%). CAQEM on one side were associated with a lower VOR gain on the contralateral side (p<0.004) and helped distinguish Menière’s disease from vestibular migraine (p = 0.01).

**Conclusion:**

CAQEM are a common phenomenon, most likely caused by a saccadic/quick phase mechanism due to gain asymmetries. They could help differentiate two of the most common causes of recurrent vertigo: vestibular migraine and Menière’s disease.

## Introduction

Patients with a deficient vestibulo-ocular reflex (VOR) cannot stabilize their gaze during head impulse testing. They re-fixate the target with compensatory saccades, i.e., quick eye movements in the opposite direction of those of the head [1]. Overt saccades, which occur after the head movement, can be detected during clinical examination [2]. Covert saccades, which occur during the head movement, require search-coil or video head impulse testing (vHIT [3]) to be observed.

**Figure 1:**
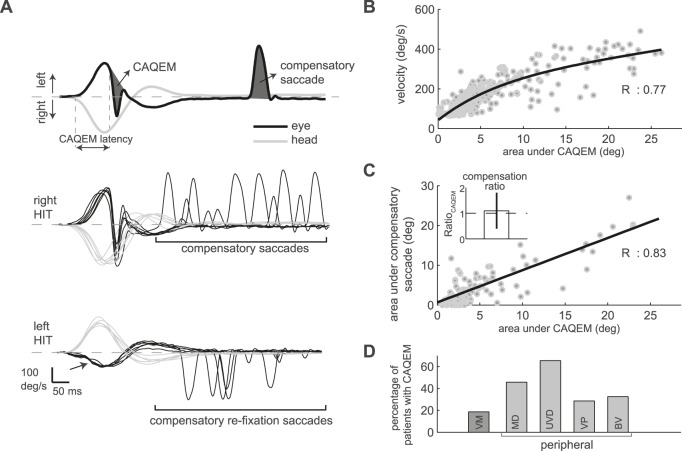
Example, saccade main sequence, compensation and clinical diagnoses. A: Top panel shows representative eye (black) and head (gray) velocity traces during head impulse testing (HIT). The covert anti-compensatory quick eye movement (CAQEM) during the head movement has a latency of 96 ms. The CAQEM is compensated for by a saccade (dark gray areas within the CAQEM velocity curve and that of the compensatory saccade match). Middle and bottom panels show eye and head traces for right and left HIT from one subject. CAQEMs on the right side are compensated for by saccades. There is a vestibulo-ocular reflex (VOR) gain deficit on the left side (note how eye velocity does not match head velocity, arrow) with compensatory re-fixation saccades toward the target. B: CAQEM peak velocity as a function of amplitude (area under the curve) for all CAQEMs from all subjects. An exponential fit (black line) shows that CAQEMs follow the saccade main sequence. C. Regression line (black) between the area under the saccades plotted against the area within the corresponding CAQEM indicates that saccades are compensatory (each dot represents the mean of one subject). Inset shows the mean compensation ratio (Ratio_CAQEM_ and SE) calculated by dividing the area under the compensatory curve by that of the CAQEM. D. Percentage of patients with CAQEMs distributed for the different clinical diagnoses established by neuro-otological experts according to current standards: vestibular migraine (VM), Menière’s disease (MD), unilateral peripheral vestibular deficit (UVD), vestibular paroxysmia (VP), and bilateral vestibulopathy (BV). The percentage of patients with CAQEMs is higher in peripheral vestibular disease (gray), e.g., Menière’s disease, than in vestibular migraine (dark gray). UVDs have the highest percentage of CAQEMs.

In addition to these well-known compensatory saccades in VOR-deficient patients, covert anti-compensatory quick eye movements during the head movement (CAQEM, Fig. 1A) were recently observed in older individuals during vHIT [4]. CAQEM are eye movements made in the direction of the head movement, which remove the eyes from the target. Here we characterize this form of quick eye movement. On the basis of our clinical observation, we hypothesized that 1) a compensatory saccade that returns the eyes to the target after CAQEM could be mistaken for a peripheral vestibular sign during clinical examination; 2) CAQEM are associated with a peripheral vestibular deficit contralaterally and thus could be used to differentiate peripheral vestibular diseases, e.g., Menière’s disease, from vestibular migraine.

## Methods

### Ethics Statement

No written informed patient consent was obtained, because the data were analyzed retrospectively and anonymously. Munich University’s Medical Faculty Ethics Committee specifically waived consent and approved the study, which was performed in accordance with the Declaration of Helsinki.

### Patients, Setting, Data Acquisition

The data of 266 patients (aged 48±22 years, mean±SD, range 6–87 years, 48 patients under 18 years of age, 172 patients aged 18–70 years, 46 patients older than 70 years, 115 men) who presented electively to our tertiary vertigo center and were assessed with the clinical head impulse test (HIT) and vHIT were retrospectively analyzed. Clinicians had diagnosed the patients according to current clinical standards [5], [6], [7], [8], [9]. Forty-three patients were diagnosed with probable vestibular migraine/vestibular migraine, 35 patients with possible/probable/definite Menière’s disease, 37 patients with complete/incomplete bilateral vestibulopathy, 14 patients with vestibular paroxysmia, 26 patients with a unilateral peripheral vestibular deficit, and 111 patients with other diagnoses (including somatoform as well as multifactorial dizziness). Neuro-otological experts had rated and documented the clinical HIT [1] prior to the vHIT examination. Different examiners had performed vHIT and clinical HIT. The data were analyzed by a third person independently of the clinical and vHIT exams. vHIT had been recorded with the EyeSeeCam system. In analogy to Bartl et al. [10], eye movements had been recorded with a digital video camera (sampling rate 220 Hz), head movements with inertial sensors mounted on the EyeSeeCam system (in contrast to Bartl et al. [10], only the left eye was monocularly recorded and no additional camera on a bite bar was used). Patients had been instructed to fixate a target located 3.3 m directly before them at eye level. The examiner, standing behind the patients, had delivered head impulses in the plane of the right and left horizontal semicircular canals. The mean number of vHITs was 33±21 per patient (right vHITs 17±11 and left vHITs 16±11, mean±SD). vHIT duration was 166±35 ms and covered 16±4° of horizontal rotation with a peak velocity of 179±34°/s.

### Data Analysis

Data were analyzed offline (MATLAB®, Mathworks). VOR velocity gain for left (*g_L_*) and right (*g_R_*) rotations was determined by regression analysis [11]. Gain values <0.7 were considered pathological [3]. Normalized gain asymmetry was 100*(*g_L_*−*g_R_*)/(*g_L_+g_R_*) [12]. When determining normalized gain asymmetries for bilateral vestibulopathy, we excluded patients with very small gains on both sides (g_L_+g_R_<0.1, n = 1 in our sample of patients) to avoid bias due to exaggerated asymmetry. This does not affect the results. In addition, for patients with bilateral vestibulopathy, unnormalized gain asymmetry was determined as the gain difference (g_L_−g_R_) between both sides as described in Weber et al. [13]. When the sign of the asymmetry was not relevant, we used the term “absolute” as the absolute value of normalized gain asymmetry [abs(100*(g_L_−g_R_)/(g_L_+g_R_))] or unnormalized gain asymmetry [abs(g_L_−g_R_)]. HIT start was detected when head velocity first reached 20°/s. HIT end was when head velocity crossed the zero line again. CAQEM and compensatory saccades were automatically detected as quick eye movements that accelerated above 2000°/s^2^ and exceeded VOR slow phase velocity by at least 65°/s, i.e., CAQEM were defined as the movement deviating from the VOR slow phase velocity. CAQEM latency was considered the duration between HIT start and CAQEM onset (Fig. 1A). Areas within the CAQEM and under the compensatory saccades (Fig. 1A) were compared to assess whether saccades compensated for CAQEM. The compensation ratio (Ratio_CAQEM_) for each subject was calculated by dividing the area under the compensatory curve by that of the CAQEM. If multiple compensatory saccades were observed, their cumulative area was taken as the compensation. In addition, the compensation ratio was computed taking the VOR into account (Ratio_CAQEM+VOR_): the area under the compensatory curve was divided by the positional difference of eye and head movements at the end of the head impulse (i.e., the positional shortfall due to CAQEM and an imperfect VOR). For statistical analysis, one-way analysis of variance (ANOVA) was used to test the differences between groups. The one-sample t-test was used to check whether the test data had a specific mean. When normality of data was violated (assessed by the Kolmogorov-Smirnov test), non-parametric alternatives (one-sample Wilcoxon signed-rank test, Mann-Whitney U test) were used. Pearson’s chi-square test was used to assess the relationship between two categorical variables. The significance level for the statistical analysis was set at p = 0.05.

## Results

At least one covert anti-compensatory quick eye movement was observed in 100 of the 266 patients (38%; see Fig. 1A for example). CAQEM were equally common in different age groups (50% of children, 35% of patients aged 18–70 years, 33% of patients older than 70 years; no age differences according to Pearson’s chi-square test, p = 0.14). The mean CAQEM occurrence rate (per HIT trial) was 11±10% (mean±SD), and latency was 83±30 ms. CAQEM followed the saccade main sequence characteristics [14] (Fig. 1B).

After CAQEM, 91% of patients performed saccades in the opposite direction. Figure 1C shows that saccades compensated for CAQEM. The mean compensation ratio (Ratio_CAQEM_) was 1.1±0.7 (no difference to one, two-tailed t-test, p = 0.21). Assessing how compensatory saccades accounted for both CAQEM and VOR (see Methods) revealed a compensation ratio (Ratio_CAQEM+VOR_) of 0.84±0.77, mean±SD (between 0.5 and 1, 0.65/0.35/1.0, median/lower/upper quartiles, one-sample Wilcoxon signed-rank test on the difference to 0.5/0.75/1, p = 0.001/0.46/0.001). Compensatory saccades did not lead to more false pathological clinical HIT assessments. The specificity of the clinical HIT with respect to vHIT gains (<0.7 was considered pathological [3]) was 85% on the non-CAQEM side and 87% with CAQEM (sensitivity was 72% and 56%, respectively).

CAQEM on the right side were associated with a lower gain on the left (−9±20%, i.e., *g_L_<g_R_*) and vice versa (4±14%, i.e., *g_R_<g_L_*). This indicated a gain asymmetry toward the non-CAQEM side (ANOVA, p<0.004). A total of 66% of patients with CAQEM on one side had such an asymmetry.

CAQEM were more common in peripheral vestibular diseases than in vestibular migraine (Fig. 1D; p = 0.004; p<0.001 for unilateral vestibular deficit vs. vestibular migraine, p = 0.01 for Menière’s disease vs. vestibular migraine, Pearson’s chi-square test). They differentiated Menière’s disease from vestibular migraine (p = 0.01, Pearson’s chi-square test, Table 1) with a sensitivity/specificity/accuracy of 46/81/65%. In comparison, vHIT gain assessment alone did not differentiate Menière’s disease from vestibular migraine (p = 0.16, Table 1) with vestibular migraine patients not having VOR gain deficits (0.96±0.12, mean of subjects±SD) or absolute gain asymmetries (5±4%, mean of subjects±SD) and patients with Menière’s disease only tending to have lower VOR gains (0.89±0.18; p = 0.05, Mann-Whitney U test; absolute gain asymmetries 9±11%). Combining VOR gain and CAQEM analysis differentiated vestibular migraine and Menière’s disease with high specificity (100%, accuracy of 60%, Pearson’s chi-square test p = 0.02, Table 1). Patients with complete or incomplete bilateral vestibulopathy (gain 0.54±0.28, mean of subjects±SD) had a normalized absolute gain asymmetry of 18±21% (bigger than 10%, one-sided t-test, p = 0.02, n = 36) and an unnormalized absolute gain asymmetry of 0.14±0.17 (bigger than 0.075, one-sided t-test, p = 0.01, n = 37). 33% of them displayed CAQEM (Fig. 1D).

**Table 1:** Differentiating Menière’s disease and vestibular migraine using combinations of gain deficit, gain asymmetry and occurrence of covert anti-compensatory quick eye movements (CAQEM).

	Sensitivity	Specificity	Accuracy	P-value
**CAQEM**	46%	81%	65%	**0.01**
**GAIN**	17%	93%	59%	0.16
**ASYM**	17%	81%	53%	0.87
**CAQEM+GAIN**	11%	100%	60%	**0.02**
**CAQEM+ASYM**	11%	98%	59%	0.10
**GAIN+ASYM**	11%	95%	58%	0.26
**CAQEM+GAIN+ASYM**	9%	100%	59%	**0.05**

Table 1 shows sensitivity/specificity/accuracy of distinct combinations of CAQEM occurrence, gain deficit (GAIN, vestibulo-ocular reflex gain<0.7) and gain asymmetry (ASYM, >8%) to differentiate Menière’s disease from vestibular migraine. P-values (Pearson’s chi-square test) show whether there is a relationship between the “Menière’s disease vs. vestibular migraine” differentiation and the corresponding measure (i.e., CAQEM, GAIN, ASYM and combinations). CAQEM are helpful for distinguishing Menière’s disease and vestibular migraine (see bold p-values).

## Discussion

CAQEM were commonly observed during vHIT of elective patients in our tertiary vertigo center. They were followed by compensatory saccades toward the target. However, compensatory saccades after CAQEM did not increase the rate of false pathological clinical HITs. This was unexpected, since, with the naked eye, clinicians should not be able to tell whether overt catch-up saccades compensated for CAQEM or for a VOR gain deficit. The reason for this might be that neuro-otology experts had rated the clinical test. Experts tend to accept borderline HITs as normal [15]. The high specificity and low sensitivity of HITs with respect to vHITs in our study reflect this tendency. CAQEM and consequent catch-up saccades only occurred in about every 10^th^ trial. Experts might have discarded these single pathological head impulses.

CAQEM latency [2] and amplitude-velocity relationship [14] point to a saccadic/nystagmus quick phase mechanism. This is in contrast to abrupt eye velocity declines in the direction of the passive head movement, which do not display saccade characteristics [16]. Saccades in the direction of the head movement were previously reported during passive head movements [17]. However, in that study, which used lower accelerations (∼600°/s^2^ vs. above 2000°/s^2^ in the present study), saccades in the direction of the head movement preceded VOR responses, possibly due to the instruction “to stare blankly ahead into the darkened room”. Therefore, the mechanism triggering these saccades most likely differs from that of CAQEM, which occur on top of a VOR mechanism (patients were instructed to fixate a point straight ahead). This is also supported by the differences between saccade latencies observed in that previous study and in our study (140±85 ms vs. 83±30 ms, respectively).

We suggest that CAQEM are caused by a small vestibular deficit on the contralateral side, resulting in both a gain asymmetry and a vestibular tone imbalance. In our study, CAQEM on one side were associated with a lower gain on the other side. Importantly, as recordings always assessed the left eye, the observed gain asymmetries toward both sides cannot simply be explained by a systematic gain asymmetry toward the side of the analyzed eye as observed by Weber et al. [18]. This imbalance is increased by the vestibular input from contralateral head impulses, which generates an anti-compensatory quick phase, similar to the mechanism of nystagmus [19]. Instructions may contribute to CAQEM occurrence as suggested by the relatively high frequency in children. However, there was no significant age effect.

The idea that a vestibular imbalance plays a role is strongly supported by the predominance of CAQEM in patients with unilateral vestibular deficits. CAQEM occurrence in patients with bilateral vestibulopathy is in accordance with this theory, too, because these patients also had an absolute gain asymmetry.

In summary, CAQEM during head impulses, which have saccade characteristics, might be an indication of small gain asymmetries and could help differentiate vestibular migraine from Menière’s disease, where clinical signs are sometimes ambiguous [20].
